# The Importance of Measuring Troponin in Chronic Obstructive Pulmonary Disease Exacerbations: A Systematic Review

**DOI:** 10.7759/cureus.17451

**Published:** 2021-08-26

**Authors:** Khaled A Elmenawi, Vishwanath Anil, Harpreet Gosal, Harsimran Kaur, Hyginus Chakwop Ngassa, Lubna Mohammed

**Affiliations:** 1 Surgery, California Institute of Behavioral Neurosciences & Psychology (CIBNP), Fairfield, USA; 2 Internal Medicine, California Institute of Behavioral Neurosciences & Psychology (CIBNP), Fairfield, USA; 3 Internal Medicine, Emergency Medicine, California Institute of Behavioral Neuroscience & Psychology (CIBNP), Fairfield, USA; 4 Family Medicine, California Institute of Behavioral Neurosciences & Psychology (CIBNP), Fairfield, USA

**Keywords:** pulmonary exacerbation, pulmonary and cardiac pathophysiology, smoking tobacco, copd exacerbation, copd: chronic obstructive pulmonary disease, cardiac troponin, obstructive lung diseases, serum biomarkers, cardiac troponin i, elevated troponin

## Abstract

Chronic obstructive pulmonary disease (COPD) is one of the leading causes of death worldwide. Many patients with acute exacerbations need intensive care. There are many cardiovascular risk factors and comorbid conditions linked with COPD. Troponin elevation is used for the diagnosis of myocardial infarction. However, it is commonly elevated in patients with COPD. This systematic review followed the Preferred Reporting Items for Systematic Reviews and Meta-Analyses (PRISMA) guidelines. PubMed and Scopus were searched for relevant articles. A total of 383 papers were identified. Out of the 383 papers, only 30 papers remained for final synthesis after removing duplicates, screening for relevant articles, and assessing eligibility criteria. After the quality appraisal, 11 papers were chosen for inclusion in this study. COPD is characterized by obstruction and inflammation of the airways. It is caused mainly by smoking, by causing harmful changes in the structure of the airways. It usually presents with dyspnea, cough, and/or production of sputum. Troponins are regulatory proteins found in the myocardium and skeletal muscles. The cause of its elevation in COPD and acute exacerbated chronic obstructive pulmonary disease (AECOPD) remains unclear. However, several reasons and factors have been suggested. The most intriguing fact is that high troponin in COPD, especially in exacerbations, has been linked in many articles to a higher risk of death. Furthermore, it could serve as a tool for better assessment and management of COPD patients. We found that troponin predicts death and poor outcomes in COPD and AECOPD. The exact mechanism of its elevations is not clear. We believe it can be a valuable tool for clinicians in better managing COPD and assessing the severity of the exacerbations.

## Introduction and background

Chronic obstructive pulmonary disease (COPD) is one of the leading causes of mortality and morbidity in the world. It is ranked the fourth cause of mortality worldwide, representing a challenge to public health that can be prevented and treated. Furthermore, the World Health Organization (WHO) predicts that COPD will become the third leading cause of death worldwide by 2030 [1]. Most of the exacerbations are caused by infections with viruses or bacteria. However, up to a third of exacerbations do not have a known cause [2]. Many patients with COPD get admitted to the intensive care unit (ICU) during acute exacerbation [3]. The exacerbations are events that occur in the natural course of COPD outside the normal range of the usual day-to-day variations. They are acute in onset and may justify changing the usual medications in COPD patients [1]. The literature is rich with information implicating cardiovascular system (CVS) abnormalities as major risk factors in COPD prognosis [2]. The comorbidity of cardiovascular disease (CVD) in COPD patients is dominant and probably the most common and most crucial coexisting illness due to the shared risk factors such as the male sex, age, smoking [1]. Pulmonary hypertension, right ventricular dysfunction, dysrhythmias, and ischemic heart diseases are famous complications of COPD. Recently, it has been shown that in COPD patients, dysfunction of the left ventricle has a negative impact on tolerance to exercise, being concomitant with anxiety and depression, low carbon monoxide (CO) diffusion, and an increase in the prevalence of right ventricular dysfunction [4]. Cardiac troponin T (cTnT) and cardiac troponin I (cTnI) function as regulators of calcium in the interaction of actin and myosin. Cardiac troponin T has not been recognized outside the cardiac cells. However, it is expressed in small quantities in skeletal muscle. Elevated cardiac troponin is now recognized as the number one biomarker in diagnosing myocardial infarction [5]. It was believed that troponin is specific for cardiac disease, but it has been found that serum troponin has prognostic value in a wide spectrum of acute conditions [6]. High levels of high sensitivity (hs)-cTnT are found commonly in stable COPD patients who do not have cardiovascular disease (CVD) and are associated with higher mortality which is not related to COPD severity and other cardiovascular risk factors [7]. Also, a good number of patients hospitalized for acute exacerbated chronic obstructive pulmonary disease (AECOPD) have elevated levels of troponin [8]. The cTnI prevalence in patients with AECOPD has been reported in several studies ranging from 16.5% to 74%, with a mean of 46% [6]. One study showed that cardiac biomarkers remained high for up to five weeks after an exacerbation, while other papers report levels of cardiac biomarkers decreased gradually after an exacerbation within days to a few weeks [9]. In a study conducted at Waikato Hospital, a large regional hospital in the central North Island of New Zealand, there wasn’t enough evidence to make the diagnosis of angina or myocardial infarction in most AECOPD patients with raised troponin. This suggests that high troponin levels in exacerbations of COPD could be misleading [10]. This systematic review will discuss the utility of troponin measurement in assessing the severity of COPD, possible causes for troponin elevation in COPD besides the known myocardial infarction, prognosis, and highlight the different risk factors related to high troponin in COPD. This could help clinicians in better managing and diagnosing COPD exacerbations.

Methods

Protocol

This review article followed the Preferred Reporting Items for Systematic Review and Meta-Analyses (PRISMA) guidelines [11].

Inclusion/Exclusion Criteria

Inclusion criteria were all studies in the English language, all types of studies were included. The studies were collected from inception to March 22, 2021. Studies mentioning a relationship between troponin and COPD patients were only included, and patients were included with no regard to age, gender, sex, or ethnicity. Exclusion criteria were studies that are not in the English language, studies not related to the question of the review, editorials, and letters to the editors.

Search Strategy 

A detailed search of PubMed, PubMed Central, Medical Literature Analysis and Retrieval System Online (MEDLINE), and Scopus was conducted, and the search results were imported to EndNote Web (Clarivate, Philadelphia, USA). PubMed was searched using the keywords: (troponin) AND [(COPD) OR (exacerbated COPD)] OR (acute exacerbated COPD) which yielded 175 papers. And using Medical Subject Headings (MeSH) keywords - ("Troponin"[MeSH]) AND "Pulmonary Disease, Chronic Obstructive"[MeSH] - yielded 63 papers. Scopus database search within titles, abstracts, and keywords using the words 'Troponin AND COPD' yielded 145 papers.

A total of 383 papers were identified and imported to EndNote Web. Duplicates were removed by EndNote and checked manually by the author, leaving only 238 papers. The results of each search are shown in Table 1.

**Table 1:** Table shows the results of databases search COPD: Chronic obstructive pulmonary disease

KEYWORDS	DATABASE	SEARCH RESULTS
(troponin) AND [(COPD) OR (exacerbated COPD)) OR (acute exacerbated copd)	PubMed	175
("Troponin"[MeSH]) AND "Pulmonary Disease, Chronic Obstructive"[MeSH]	PubMed	63
Troponin AND COPD	Scopus	145

Study Selection

**Figure 1:**
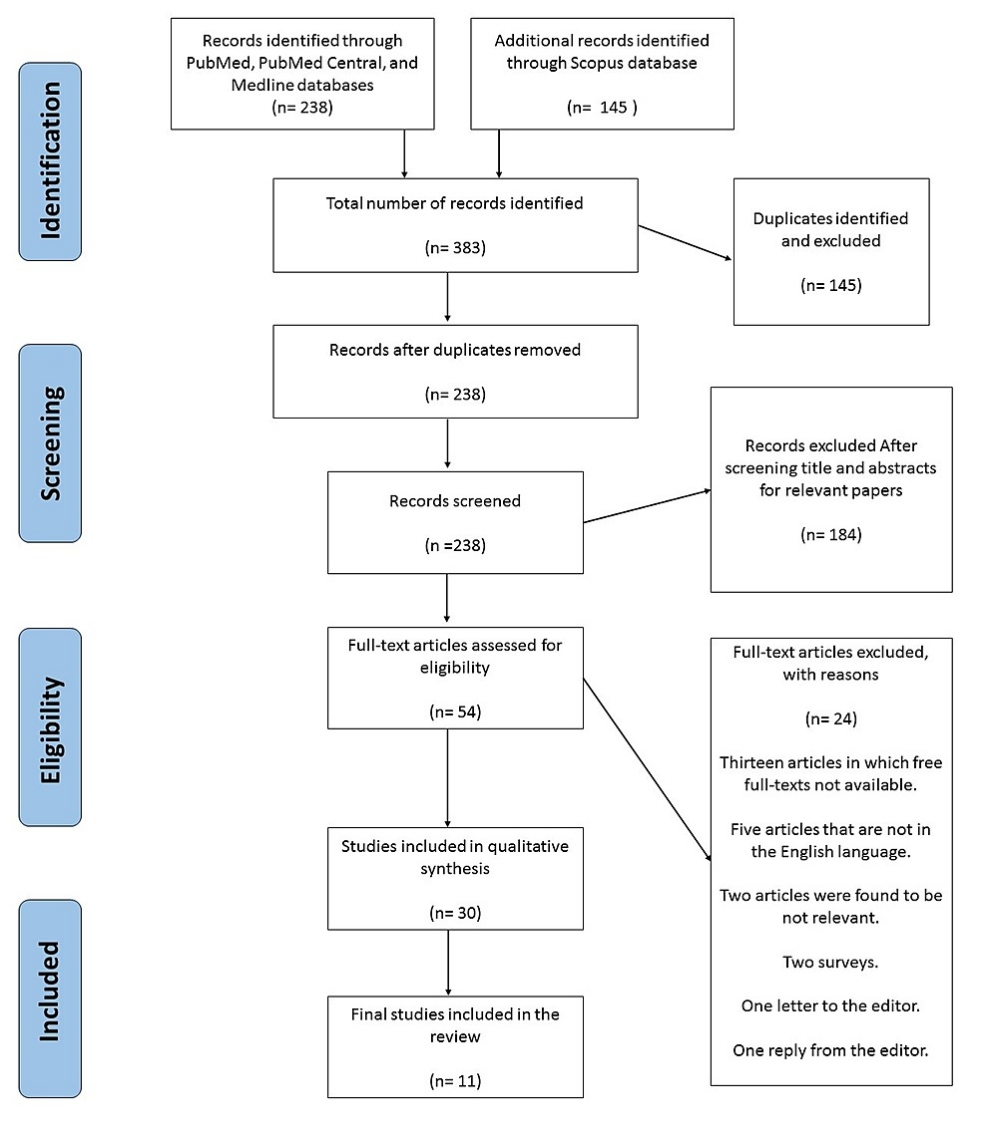
PRISMA flow chart PRISMA: Preferred Reporting Items for Systematic Reviews and Meta-Analyses

After duplicates removal, a total of 238 papers were screened for title and abstracts identifying 54 papers that are relevant to the review question. Of these papers, 24 were excluded based on reasons shown in the PRISMA diagram (Figure 1). After the quality appraisal, only 11 papers were analyzed in this review.

Quality Assessment

Critical appraisal was performed independently by the first and second authors manually, and three papers were excluded.

Results

A total of 383 papers were identified by searching the databases. After duplicates were removed, 238 papers remained, of which, 184 papers were excluded for being irrelevant. The remaining 54 papers were assessed for eligibility criteria. Twenty-four papers were removed. Thirty papers were included in the final synthesis. Quality assessment of these papers was performed by the first and second authors independently. After the quality appraisal, 27 were of good quality. The papers chosen for this review included five prospective studies, two retrospective studies, two cross-sectional studies, one systematic review, and one comparative study. Included studies are shown in Table 2.

**Table 2:** Table shows studies included in final data synthesis ECG: Electrocardiogram, AECOPD: Acute exacerbated chronic obstructive pulmonary disease, COPD: Chronic obstructive pulmonary disease, cTnT: Cardiac troponin T, cTnI: Cardiac troponin I, ICU: Intensive care unit, Hs- cTnT: High sensitivity-cardiac troponin T, BNP: Brain natriuretic peptide

Author	Year	Study design	Location	size	Conclusion
Shafuddin et al. [12]	2019	Prospective cohort	New Zealand	N=423	Cardiac biomarkers are complementary to ECG and chest radiographs in the diagnosis of acute cardiac dysfunction during AECOPD.
Shafuddin et al. [8]	2018	Prospective cohort	New Zealand	N=176	The rise of brain natriuretic peptide (BNP) continues after admission to the hospital for AECOPD, and a minority of patients have clinically significant troponin rises. The elevations in cardiac biomarkers were associated with nebulized beta₂ agonists. Suggesting that a high dose of beta₂ agonists could increase cardiac dysfunction in COPD patients.
Neukamm et al. [9]	2016	Prospective cohort	Norway	N=275	Elevated cTnT is commonly present in stable COPD without overt coronary vascular disease, and is associated with higher mortality, irrelevantly to the severity of COPD and other cardiovascular risk factors.
Noorain et al. [1]	2016	Prospective cohort	India	N=50	A significant subset of patients with AECOPD has elevated cTnI. Elevation of cTnI is associated with the need for ICU care and ventilator support. However, it is not predictive of in-hospital death. Thus, it can be used as a marker for the identification of high-risk patients during AEOPD.
Buchan et al. [2]	2015	Systematic review	N\A	N=14 studies	A link may be present between elevated BNP and cardiovascular mortality during COPD exacerbations, although the current data available are not conclusive. Due to the inconsistency in measurement, more analysis is needed.
Høiseth et al. [13]	2014	Prospective cohort	Norway	N=83	Among patients with hs-cTnT above the 99th percentile, 82% had a dynamic pattern, as requested to diagnose myocardial infarction. Stable and moderate elevations of cTnT during AECOPD are associated with worse long-term outcomes compared to patients with a dynamic pattern of cTnT.
Kelly et al. [6]	2013	Retrospective cohort	Australia	N=252	COPD patients with elevated cardiac troponin I have a higher risk of death after discharge.
Søyseth et al. [7]	2013	Cross-sectional	Norway	Index=50 Reference=124	Higher hs-cTnT is associated with AECOPD compared with stable COPD. Hs-cTnT is related to the severity in stable COPD patients. The study was not able to recognize significant determinants of hs-cTnT in AECOPD.
Høiseth et al. [14]	2011	Comparative study	Norway	N=99	Elevated cTnT is common during exacerbations and is associated with higher mortality. This positive association is more substantial in patients with tachycardia.
Brekke et al. [15]	2009	Cross-sectional	Norway	N=441	Many factors are associated with cTnT rise, probably reflecting the wide range of comorbid conditions seen in COPD. A positive association between cTnT and neutrophils is compatible with the concept that myocardial injury during an exacerbation is predisposed to an increase in the inflammatory response.
Harvey et al. [10]	2004	Retrospective review	New Zealand	N=375	Troponin rise is common during AECOPD, and it reflects the severity of the exacerbation. In most patients, there is not enough evidence to diagnose acute coronary syndrome.

## Review

Pathophysiology of COPD

**Figure 2:**
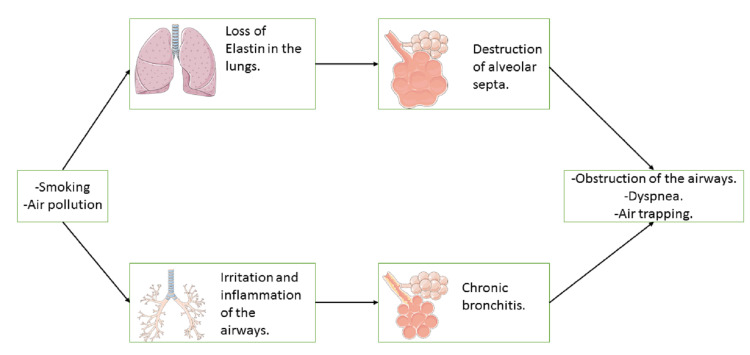
The figure shows the pathophysiological process of COPD COPD: Chronic obstructive pulmonary disease

Chronic obstructive pulmonary disease (COPD) is defined by the Global Initiative for Chronic Obstructive Lung Disease (GOLD) as a disease characterized by limitations of airflow which is not fully reversible [16]. COPD is characterized by obstruction of the airflow and abnormal lung inflammation caused by responses of the innate and adaptive immunity to the prolonged exposure to harmful gases and particles, especially smoking [17]. There is an increased number of neutrophils, macrophages, and T lymphocytes in the lungs. The release of cytokines and mediators by these cells plays a role in the disease process [17]. Furthermore, hypersecretion of mucous, ciliary dysfunction, gas exchange abnormalities, pulmonary hypertension, and systemic changes also occur in COPD [17]. The disease is slowly progressive with a long asymptomatic period, in which the lung function continues to decline [16]. The main risk factor for COPD is smoking. However, one out of six Americans with COPD never smoked before. Exposure to chemical fumes, dust, and other irritants accounts for 10 to 20% of patients [16]. Smoking contains toxic chemicals, including reactive oxygen species (ROS) or oxygen-derived metabolites. Accumulation of ROS in excess leads to harmful modifications in proteins, lipids, and deoxyribonucleic acid (DNA) [18]. The harmful changes result in higher resistance to airflow in the small airways, higher lung compliance, trapping of air, and progressive obstruction [17]. Airways become fibrotic, and the lung loses its elasticity [19]. Hypoxia develops in the late stages of COPD [16]. The inflammation and the change in pathology increase with the severity of the disease and continues despite cessation of smoking [17]. Patients with COPD can inhale a high volume of air, but they may exhale only part of that volume, resulting in trapping of air and hyperinflation [19]. When patients exercise, they have a shorter time for exhaling and less space to breathe in [19]. COPD patients usually present with shortness of breath, cough, and/or sputum production [19]. The pathophysiological process of COPD is shown in Figure 2. 

Importance of measuring troponin

Troponin is part of the myocardium and the skeletal muscles [20]. Troponin is formed of different regulatory proteins (troponin C, troponin I, and troponin T). The ones that are unique to the cardiac muscles and thus are very useful in detecting cardiac injury are troponin I and troponin T [21]. The high sensitivity (hs) troponin assays have higher sensitivity than the conventional assays. Hs-troponin assays can detect levels that are ten-fold lower than the traditional assays [21]. Non-coronary-related diseases have been reported to increase the level of plasma troponins, such as renal impairment, heart failure, pulmonary embolism, myocarditis, and AECOPD [20]. Previous retrospective studies of patients with AECOPD revealed that 18 to 70% had troponin elevation measured by conventional assay [14].

Factors contributing to troponin elevation in COPD

The mechanism of elevation still is unknown [1,10]. The elevated cTnT could possibly be the result of an inflammatory effect upon the cardiac cells [7]. A high WBC count was found in several studies associated with increased troponin levels [7,8,14,15]. COPD is characterized by major airway neutrophil inflammation, which is more pronounced during exacerbations both systemically and locally [15]. The inflammation could be increasing the progression and worsening of silent atherosclerosis [7]. Possible mechanisms for troponin elevation by neutrophils include the release of inflammatory mediators, aggregation and vascular obstruction, and oxidative stress on vessels [15]. In patients with AECOPD, Høiseth et al. found higher creatinine levels were associated with troponin T elevation [14]. A prospective study reports that patients with troponin T levels of 14 ng/L or more had a lower glomerular filtration rate (GFR) than those with troponin levels below 14 ng/L [7]. Brekke et al. studied determinants of cardiac troponin elevation in AECOPD patients, and they found that increasing creatinine had a significant independent association with high cTnT [15]. In a cross-sectional study, it is noted that hs-cTnT increases in patients with increasing creatinine in stable COPD patients [8]. In a study published in 2007 in which 615 patients had elevated troponin, 41% of cases were not caused by acute coronary syndrome (ACS), 5% of these were attributed to renal failure [22]. The mentioned papers suggest that cardiac troponin elevation in COPD patients could be caused by decreased renal clearance. It is worth mentioning that one study noted that impaired renal clearance was unlikely to be the cause of cardiac biomarkers elevation in AECOPD [9]. Several cardiovascular findings have been linked to a higher troponin level in patients with COPD [8,15]. A prospective study of 50 patients admitted to the hospital with AECOPD found that the group of patients with positive cTnT had more prevalent cardiovascular comorbidities. Left ventricular dysfunction and dilated right side of the heart were also more common among the positive group [1]. Several studies mentioned that hypoxia and tachycardia could increase the troponin release by causing ischemic cardiac injury to the myocardium as a result of the mismatch between supply and demand [6,10,12,14,15]. This is supported by finding low levels of hemoglobin to be associated with high troponin in patients with AECOPD [13,15]. Previous studies have shown that there is an increase in arterial stiffness in patients with AECOPD [9]. One study excluded hypoxia as a possible cause for troponin elevation during exacerbations [8]. In a study investigating the associations between ECG and chest radiographic findings with biomarkers of cardiac dysfunction, high troponin was best indicated by the presence of tachycardia and ischemia on ECG [12]. Other ECG findings associated with high troponin levels during exacerbation are conduction block, ST-segment depression, and T wave changes [10]. Cardiomegaly was reported to be more common in AECOPD patients with increased cTnT [23]. Pulmonary hypertension and right ventricular strain have been mentioned as potential mechanisms for troponin elevation in COPD patients [6,7,10,14,23]. Left ventricular dysfunction has been shown to be coexisting with high levels of troponin T [1,2]. Pulmonary embolism [6,19,23], and undiagnosed coronary heart disease [9,14], could also increase troponin during exacerbation in COPD. 

Predicting the outcome of COPD using troponin

In a systematic review of 14 studies, troponin was associated with mortality [2]. The mortality measured in the studies ranged from in-hospital death to death after discharging the patients. They also mention that troponin elevation is significantly related to recurrent hospitalization and period of hospitalization [2]. Kelly et al. also found elevated troponin during COPD exacerbations are associated with in-hospital mortality [6]. This was found to be not true by Noorain et al. [1], and Raji et al. [24]. Høiseth et al. followed 99 patients hospitalized for AECOPD for a median period of 1.9 years and concluded that mortality was related to elevated hs-cTnT, especially in the presence of tachycardia [14]. In a double-blinded randomized controlled trial studying the relation between cardiac troponin I levels and cardiovascular events in COPD patients, troponin I elevation was a strong and specific predictor of the occurrence of cardiovascular events and death [25]. However, mortality was also reported to be associated with high troponin independent of cardiovascular risk factors [7]. The mortality rates in COPD patients with elevated troponin differed between the studies. Høiseth et al. reported the mortality rate to be 4.6, 30.2, 58.3 per 100 patient-years in patients having hs-cTnT <14.0, 14-39.9, and ≥40 ng/L respectively [14]. Nuekamm et al. report mortality rates of 2.8, 4.4, and 11 per 100 patient-years with hs-cTnT levels of <5.0, 5.0-15, and ≥14 ng/L, respectively [7]. A prospective study compared the stable and dynamic elevations in hs-cTnT during AECOPD. Mortality rates were 113 and 27 per 100 patient-years, respectively [22]. Shafuddin et al. concluded that patients requiring hospitalization during exacerbation had a higher increase in cardiac biomarkers compared to those who were managed in the community [8]. The need for ICU care in COPD patients is also associated with elevated troponin [1,24]. Not only troponin rise but also the pattern of elevation could predict the outcomes. Høiseth et al. showed that AECOPD patients with a stable rise in troponin had worse outcomes than those with a rise/fall pattern of troponin elevation [13].

Clinical value of measuring troponin during COPD exacerbations

Assessing troponin levels in AECOPD patients helps in identifying those who are at higher risks for poor outcomes [1,8]. This could help in the proper management of these patients for better outcomes [1,6]. Shafuddin et al. revealed that cardiac biomarkers are helpful diagnostic tools for the detection of acute cardiac dysfunction during COPD exacerbations. The study showed that routine ECG and chest radiographs were insufficient for excluding cardiac dysfunction during AECOPD, proposing that there should be a low threshold for investigating cardiac function during COPD exacerbations [12]. Abroug et al. studied the accuracy of cardiac biomarkers in the diagnosis of left ventricular dysfunction in AECOPD, and troponin T was shown to have a significant value in ruling in/out left ventricular dysfunction during exacerbations of COPD [26].

Limitations

The main limitation is the relatively small number of patients included in the studies. Also, in some patients, troponin measurements were performed based on the decision of the available clinician at the time of presentation. Also, different time points were studied in the included papers. Hence, further large studies should be conducted for different time points. It is worth mentioning that the studies could have missed acute coronary syndrome in patients with elevated troponin, interfering with the suggested causes of elevation in the studies. How the included studies defined COPD and AECOPD were not the same throughout the papers. This could lead to the false inclusion of patients in the studies. Two of the included studies are cross-sectional in design, limiting the interpretation of the cause and effect relationships in these studies. Another limitation is the relatively narrow patient demographic since most of the included studies were conducted in Norway or New Zealand. Furthermore, we limited our study to papers in the English language and free full-text papers. 

## Conclusions

Measuring troponin in COPD patients was found to predict the outcome of the patients. High troponin predicts worse prognosis and death, ranging from in-hospital death to death after discharge. The cause of elevation of troponin in COPD and AECOPD patients remains unclear. Troponin could be used to assess COPD patients along with other routine investigations to provide a better understanding of the condition of the patients. However, it is not known how troponin levels can be used as a tool for better managing patients with COPD and AECOPD. This paper will help the clinicians to understand the possible factors causing troponin elevation and how different levels of troponin predict different outcomes. Further studies are needed to identify the possible cause(s) for elevated troponin in COPD and AECPD and to study how its measurement could be used in clinical practice to better manage COPD patients, especially during exacerbations.
